# Our New York Letter

**Published:** 1887-01

**Authors:** 


### OUR NEW YORK LETTER.

PASTEUR AND HIS INOCULATIONS—THEIR PRACTICE IN NEW YORK—DR. WM. A. HAMMOND'S OPINION OF COCAINE AS EXPRESSED BEFORE THE NEUROLOGICAL SOCIETY—HIS EXPERIMENT WITH IT—THE DIET KITCHEN ASSOCIATION—ITS OBJECTS—THE HOSPITAL SATURDAY AND SUNDAY COLLECTIONS—HOW DISTRIBUTED, ETC.

*Editors Atlanta Medical and Surgical Journal:*

Although we do not hear very much just now about Pasteur and his inoculations against hydrophobia, still among the medical profession the subject is receiving some attention here. It may be fairly said that about one-half of our physicians look somewhat skeptically upon Pasteur's claims, while the other half are true believers, and among the latter are a few enthusiasts. Dr. Valentine Mott, of this city, has recently been occupied in treating three patients who were said to have been bitten by a mad-dog in Missouri. Two of the patients are the sons of a physician from that State and the other is his office boy. After cauterizing the wounds, it was the doctor's intention to take the three patients to Paris for treatment, but having learned of Dr. Mott's studies of Pasteur's method, and that he possessed some of the virus from Pasteur's rabbits, he decided to place the cases under Dr. Mott's care. Two of the boys were bitten on the leg and the other on the hand, but their wounds had not healed upon their arrival in New York. According to the practices of M. Pasteur, Dr. Mott has made daily inoculations for ten days upon these patients and this completes the treatment. There appears to be little doubt about the dog being mad. It had been bitten by another dog showing symptoms of hydrophobia, and in five days after attacking the doctor's children, it died in convulsions. Other dogs bitten by it have also exhibited signs of rabies and died. This is said to make about twelve patients treated by Dr. Mott on the inoculation plan since his return from Paris.

In speaking, before the Neurological Society, of the

"COCAINE HABIT,"

which has been attracting much attention of late, Dr. W. A. Hammond gave his personal experience with this drug. He has used the fluid extract, but finds, besides its unpleasant taste, that it is irritating to the stomach. The ordinary wines differ so in composition that he uses one containing hydrochlorate of cocaine, two grains to the pint, which he prescribes in spinal irritation with very favorable effect, and also where a tonic is indicated. When fatigued, at the end of the day, he is in the habit of drinking a wine-glassful himself. The stimulation produced is not, he says, followed by disagreeable after-effects. He also recommends it in dyspepsia with an intolerant stomach, and believes it blunts the sensibility of this organ. For this purpose he orders about half a dozen one-drachm doses at intervals of twenty minutes. The first doses may be vomited, but the last will be retained. It is better to use the salt when a more decided effect is wanted. There have lately been some very startling stories in our newspapers setting forth the terrible effects of cocaine upon those who have become victims to the "habit," but Dr. Hammond believes these popular opinions are erroneous. With a desire to know the true effect of cocaine, the doctor took a hypodermic of half gr. of the hydrochlorate. This caused a sensation of stimulation and mental happiness, followed during the night by sleeplessness for a time. Headache followed in morning and marked diuresis. The following evening he increased the injection to two grains. During the stimulation which followed, he wrote ten pages of foolscap, but afterwards found out that there was no sense in what he had written. The night after he took a hypodermic of three grains and soon became very talkative.

He was aware of what transpired, but could not control himself. It was a long while before he was able to sleep, and he awoke with a headache again. No abscesses occurred at the points of injection. After four or five days he took six grains hypodermically, and soon felt decidedly out of sorts. There was no loss of consciousness; he was rather disposed to keep quiet, though sleep was impossible during the night. The heart's action was augmented. Three nights after this he injected eight grains with no important differences in the effects. The following night he used eighteen grains and soon felt intensely enlivened, but did not remember just what happened. He suffered from headache for two days and cardiac palpitation; but he experienced none of the terrible effects which have been ascribed to this drug; no inclination to commit murder or display any violence. In one of his patients, taking from one to five grains daily for three months, there was no difficulty in discontinuing its use. In a case of opium habit, where he ordered cocaine, it took the place of opium and the patient did not form the cocaine habit. Dr. Hammond believes the so-called cocaine habit is like the tea or coffee habit, but differs from the opium habit. He denied that there was a single case in existence where the patient could not stop its use at will.

For some years there has existed in New York a society called the

"DIET KITCHEN ASSOCIATION,"

having as its main object the furnishing of nourishing food for the sick-poor. In additionto this, clothing is often provided for the needy.

The association consists of a President, Vice-President and Treasurer, and there are four of these Diet Kitchens, each being presided over by a directress. The food, consisting of milk, beef tea, beef soup, mutton broth, eggs, rice, oatmeal, corn-starch, farina and barley, is distributed to all those bringing the proper physician's requisitions.

According to their annual report of last year, there were 20,755 requisitions in the first kichen for 2,965 patients. In the second, 18,613 for 2,659 patients; in the third, 37,611 for 5,373 patients; and for the fourth, 11,481 for 1,788 patients.

These articles of food are dispensed in pints and half pounds to each patient. With the approach of winter, an increased demand has been made upon the resources of this association, and a fair has just been held in aid of this deserving charity. It proved a decided success financially and otherwise.

THE HOSPITAL SATURDAY AND SUNDAY COLLECTION

is a movement which is growing in importance and popular favor yearly. This collection is taken on the last Saturday and Sunday of each year, and the present month will witness its ninth anniversary.

There have been two fields of operation, the religious and the secular; in the former it has been attempted to have every creed represented in the work. Last year only two hundred churches contributed, but this year it is expected that many more will join. Twenty-seven hospitals receive money from this source; there is a general fund which is distributed according to the number of days of free treatment given by each hospital during the year. The money may also be designated for special hospitals, but it is more desirable that contributions be made to the general fund.

The trades will also be appealed to and the sum collected from a particular trade set apart for taking care of any patient recommended by the same. With two or three exceptions, our city hospitals depend day by day upon the gifts of the public to meet their running expenses. The funds are distributed by a committee consisting of the Mayor, Postmaster, President of the Chamber of Commerce, and several prominent citizens. Last year the total collection amounted to $46,085. The work of arrangements for the present year is well under way, and it is expected that over $50,000 will be realized through the friendly rivalry existing between religious societies and business houses.

A. F.

December, 1886.

